# Viscoelastic recovery times of chondrocytes measured using a novel 3D-printed microfluidic device

**DOI:** 10.1088/1361-6501/adf65a

**Published:** 2025-08-12

**Authors:** Michael Neubauer, Priyanka Brahmachary, Alan Fine, Ronald June, Stephan Warnat

**Affiliations:** 1 Department of Mechanical and Industrial Engineering, Montana State University, Bozeman, MT, United States of America; 2 Center for Biofilm Engineering, Montana State University, Bozeman, MT, United States of America; 3 Department of Physiology and Biophysics, Dalhousie University, Halifax, Nova Scotia, Canada; 4 Alentic Microscience Inc., Halifax, Nova Scotia, Canada; 5 Department of Microbiology and Cell Biology, Montana State University, Bozeman, MT, United States of America

**Keywords:** microfluidic device, cell mechanics, viscoelastic properties, chondrocytes

## Abstract

This paper presents the development, production, and application of a 3D-printed microfluidic device designed to measure the viscoelastic recovery time of cartilage cells, chondrocytes. Bovine chondrocytes were imaged using a confocal microscope while compressed by a movable glass plate. Their recovery was monitored by tracking their projected area over time, converting it into a linear strain, and fitting it to a Burgers mechanical model. Strains ranging from 10% to 60% were applied to the cells, and model parameters, including the viscoelastic recovery time, were derived. We found that cells subjected to strains greater than 40% exhibited radially-symmetric deformations. This radially-symmetric deformation, possibly cell blebbing, was observed as a short-term effect, with the cell fully recovering its initial shape. Non-blebbing and blebbing chondrocytes exhibited viscoelastic recovery times of 42 s and 38 s, respectively. While the recovery time did not depend on the magnitude of applied strain, the measured permanent strain increased with higher applied strain magnitude. Overall, this study demonstrates the use of a new, low-cost 3D-printed microfluidic device in combination with advanced microscopy for characterizing the viscoelastic properties of cells.

## 1. Introduction

Biological cells are protected from mechanical loading by their cytoskeleton, which plays a key role in determining the cell shape and mechanical properties [1]. Due to the diversity of environments, many different types of cells exhibit a broad range of viscoelastic properties. Many cells sense their mechanical environment and respond or adapt to it through the process called mechanotransduction [2, 3]. One example of this type of cell is chondrocytes, which are embedded in articular cartilage that lines the ends of bones in diarthrodial joints. These cells maintain the extracellular matrix, the predominant component of cartilage, and produce a pericellular matrix (PCM) shell around themselves for mechanical protection, chemical exchange, and biochemical signaling [4–7]. Changes to the metabolic state of chondrocytes have been linked to the onset of osteoarthritis (OA) [5], a common chronic disease affecting more than 300 million people worldwide and posing a significant economic burden on patients and healthcare providers [8, 9]. Measuring mechanical biomarkers provides insight into cell health and environmental adaptation, as mechanical properties are closely tied to the structural and functional integrity of cells.

Viscoelastic characterization of chondrocytes, and cells in general, is most often performed with two conventional techniques: atomic force microscopy (AFM) and micropipette aspiration (MPA). AFM involves moving a small probe in contact with a single cell; the specific force or indentation depth is precision controlled, and the cell response force is recorded [10]. In MPA, negative pressure forces cells to enter small glass pipette tips, and the cells’ deformation is imaged with light microscopy [11]. A significant drawback of these techniques is their low throughput, ranging between 1 and 10 cells h^−1^ [12]. The main reason for the low throughput is the difficulty in handling and positioning cells at the single-cell scale.

Recent advances in microfluidics offer a promising solution by enabling the rapid mechanical assessment of individual cells in controlled fluidic environments. This technology allows for precise handling and manipulation of cells at high speeds. In general, mechanical characterization approaches using microfluidics are classified as passive (static geometry) or active (dynamic geometry). In passive devices, fluid flow and optical imaging can be dynamically controlled; however, the channels remain static. Cells are deformed by contacting the walls or the fluid within which they are suspended. Active devices dynamically control fluid flow, similar to passive methods, but with the added flexibility of adjustable channel dimensions. A moving wall of the microdevice typically deforms cells.

Microfluidic MPA (MMPA) is an example of a passive devices that demonstrate throughputs of up to 100 cells h^−1^, which is typically accomplished by fabricating multiple channels in parallel within the field-of-view (FOV) of a microscope camera [13–17]. The measurement and analysis of MMPA mimics classical MPA where the measured deformation of the cells is fit to an equivalent mechanical model, such as a three-element standard linear solid (SLS) model. Scaling up the number of channels beyond roughly 20 is difficult because of the pressure drop variability of the parallel channels and the FOV of the camera [14]. A more common and often higher throughput approach is deformability cytometry (DC), which has been often used to compare cells from malignant and healthy donors [18–20]. There are three subsets within DC: constriction-DC, fluid shear-DC, and extensional flow-DC. In constriction-DC, cells are deformed by constriction channels smaller than the cells [21–23]. Compared to MMPA channels, cells are less strained and pass quickly through the constriction. Fluid shear-DC is a contactless approach, as the cells do not come into contact with the channel walls, thereby eliminating the frictional interaction between the channel walls and the cells. Instead, cells are deformed in a characteristic bullet-like shape by hydrodynamic shear stress resulting from the parabolic velocity profile of the fluid [24, 25]. Lastly, extensional flow-DC employs cross-shaped channels to deform cells hydrodynamically [26, 27]. While DC can be extended to measure the direct mechanical properties of cells, such as the Young’s modulus, many of the listed examples focus on comparing and distinguishing different cell populations based on their deformability in a particular microfluidic device by measuring constriction entrance and passage time, cell shape change, and deformation rate. Precise flow control, high-speed imaging, and single-use devices are factors that inherently limit the accessibility of these passive microfluidic methods.

Active microfluidic devices with variable-height channels, capable of compressing cells, are often made using PDMS membranes [28]. A thin membrane separates multilayer PDMS channels; the lower channel is loaded with cells, while air or liquid in the other channel is pressurized to deform the membrane and compress the cells [29, 30]. A difficulty of this method is the non-uniform deflection of the PDMS membrane. One approach to overcoming this challenge is to trap cells directly underneath the center of the membrane, ensuring that the deflection is nearly uniform [31, 32]. Ho *et al* describe the design and fabrication of a device capable of applying cyclic planar compression to single adherent breast epithelial MCF10A cells; however, no mechanical properties were extracted [31]. Yokokura *et al* compressed single osteoblast cells under a PDMS membrane and extracted the Young’s modulus of the cells via a mechanical model [33]. Du *et al* distinguished 3T3 fibroblasts and HL60 cells by their viscoelastic recovery time after compressing with a PDMS membrane [34]. Another approach is to thicken the membrane in a localized area, forming an elastomeric piston [35]. Onal *et al* adhered SKOV-3 ovarian cancer cells underneath an elastomeric piston and compressed cells with a defined pressure, finding that pressure magnitude (applied strain) impacted cell recovery time [36]. Incorporating a variable-height chamber on a CMOS imager has been achieved [37], which significantly increases FOV compared to conventional microscopy systems. Further examples of devices for applying deformation to cells are summarized in a review paper by Onal *et al* [28].

To date, passive geometry devices generally have a higher measurement throughput and simpler fabrication processes. Conversely, active geometry devices offer the capability to apply dynamic stress or strain, are able to monitor longer-term cell responses, and possess increased resistance to clogging, rendering them reusable. Active devices can accommodate cells of different sizes, whereas passive devices must be designed for a particular cell size. Owing to their more straightforward design and fabrication, there are more examples of passive devices than active devices in the literature. The throughput of passive devices has already been increased to greater than 1000 cells s^−1^, but achieving such rates requires the use of a high-speed camera, which dramatically increases both cost and complexity. Active device throughput can be improved by parallelizing the number of cells deformed by the moving wall and expanding the camera FOV.

In this study, we present the fabrication and application of a novel active-geometry microfluidic device for measuring the viscoelastic recovery time of bovine chondrocytes. Given the prevalence of OA, the mechanical properties of chondrocytes have been previously reported in the literature and remain a relevant biological question as the role of chondrocytes in OA is investigated. The viscoelastic recovery time of chondrocytes has been reported using AFM and MPA (table 1) and ranges between 20 and 50 s, but it has not been measured with a high-throughput technique. This work demonstrates the proof-of-principle of a versatile device for mechanically characterizing cells and is compatible with large FOV imaging. The device differs from typical active-microfluidic mechanical characterization methods in that it does not incorporate a PDMS membrane, instead deforming cells between two pieces of glass. The device is flexible in that it can, in principle, characterize cells ranging from 1 to 75 μm by adjusting the pressure applied to modulate the position of the glass clamping plate.

**Table 1.** Examples of previously reported viscoelastic recovery times of chondrocyte cells. *times were estimated from graph.

Chondrocytes	Method	Recovery time (s)	References
Human—healthy	MPA—3 element model	33 ± 20	[38]
Human—OA	MPA—3 element model	43 ± 34	[38]
Human—healthy	MPA—3 element model	71, 55, and 63*	[39]
Human—OA	MPA—3 element model	36, 114, 52*	[39]
Porcine—middle/deep depth	AFM—Hertz model	9.0 ± 6.2	[40]
Porcine—middle/deep depth	MPA—3 element model	37 ± 26	[40]
Human—healthy 18–35 years old	MPA—3 element model	21*	[41]
Human—55+ healthy	MPA—3 element model	22*	[41]
Human—55+ OA	MPA—3 element model	29*	[41]

## 2. Materials and methods

### 2.1. 3D printed flow cell fabrication and assembly

**Figure 1.**
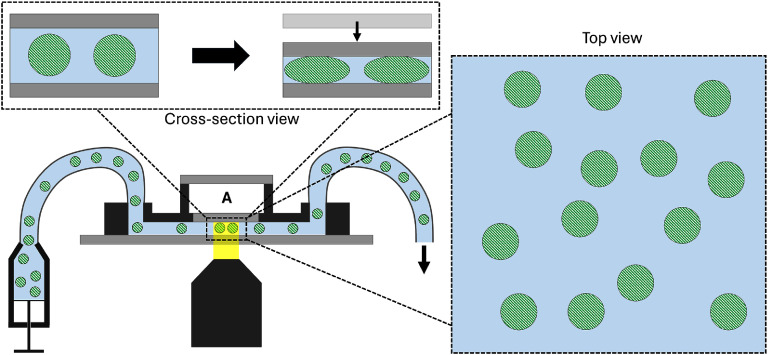
A conceptual overview of the device. Cells (striped, green circles) are suspended in a buffer solution in a syringe and flown through a 3D printed microfluidic device which is mounted in a microscope. The flow is stopped and all the cells in the field-of-view (top view) are compressed between two pieces of glass (cross-section view). After some time, the compression is removed and the apparent diameters of all the cells are imaged during their recovery. To compress the cells, the air pressure in the control chamber (labeled **A**) is adjusted with a feedback-controlled pressure system described in the Supplementary data.

The flow cell developed in this work is illustrated in figure 1. The main fluidic channel features a movable piece of glass, allowing for the creation of a variable-height channel to compress cells. Above this piece of glass is a control chamber that is pressurized to actuate the movable glass surface. All the cells below this movable glass piece are compressed between the two glass plates as the height of the channel becomes shorter than the diameter of the individual cells. The device was assembled in multiple stages, including 3D prints and silicone/glue curing steps.

**Figure 2.**
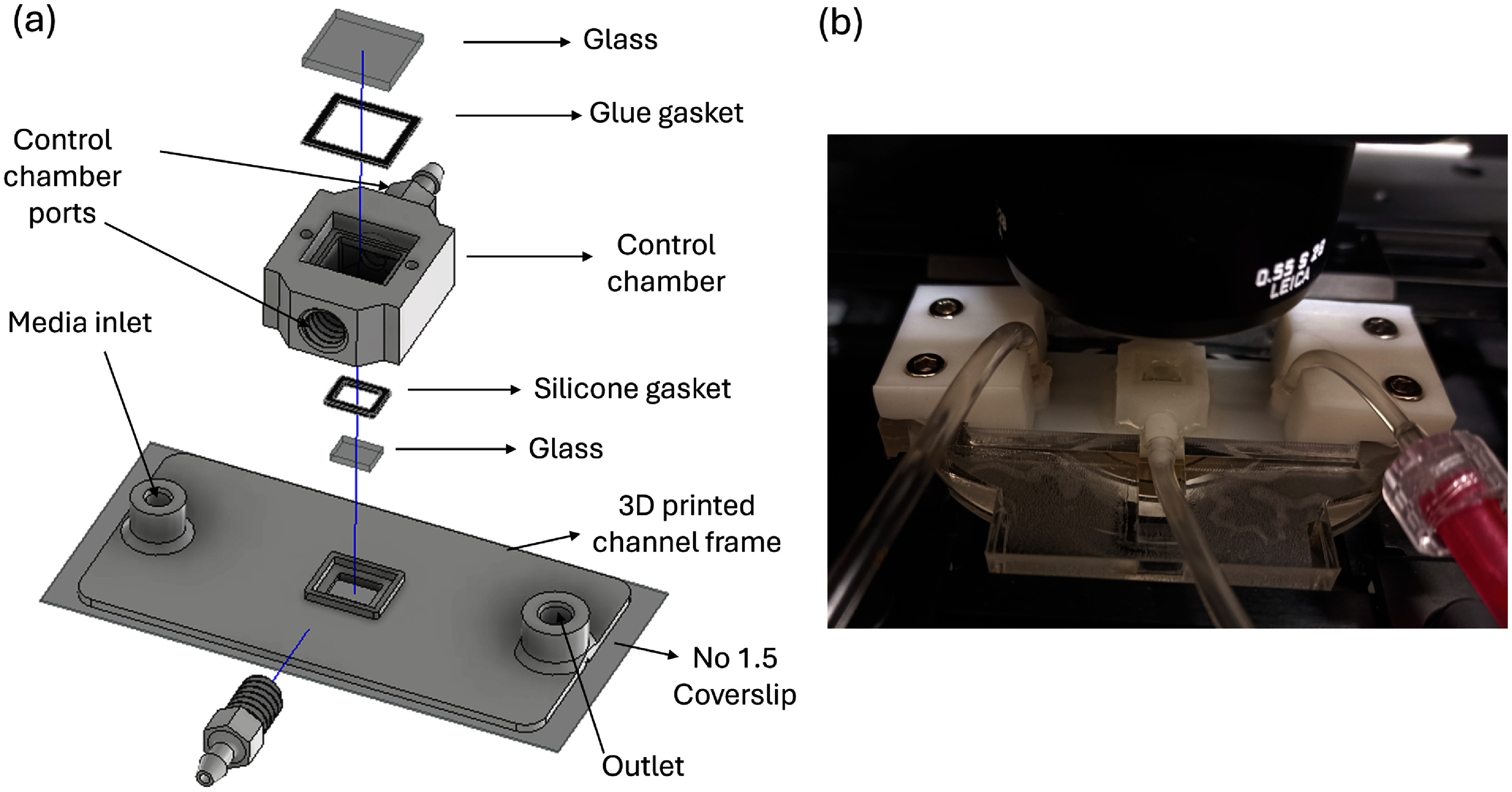
(a) Exploded view of the 3D-printed device, showing all the components of the device. (b) An image of the assembled device clamped in the acrylic flattening jig and mounted in the inverted confocal microscope.

The variable-height channel, shown in figure 2(a), was printed directly onto a No. 1.5 coverslip (24 × 50 × 0.17 mm) using an Epax X1 4k printer and eSun PLA-Pro resin. Before printing, the coverslip was treated with bind-silane (3-(trimethoxysilyl) propyl methacrylate 17-1330-01; GE Healthcare, USA) to improve the adhesion of the resin to the coverslip, as described in [42]. A coverslip is necessary as it improves imaging resolution since microscope objectives are commonly corrected for use with No. 1.5 coverslips. A challenge of printing on such a thin piece of glass is that the resin shrinks when cross-linked, causing the thin coverslip to deform. To mitigate this, the 3D-printed structure was designed to be as thin as possible (∼1 mm) to reduce the internal stress in the resin, and the coverslip was reinforced by a microscope slide (25 × 75 × 1 mm) connected to the coverslip with thermal-release-doubled-sided tape (RevAlpha 319Y-4M, Nitto, USA). The microscope slide, tape, and coverslip stack were attached to the build plate of the 3D printer with double-sided polyimide tape (Kapton, DuPont, USA), and the thickness of the stack was accounted for in the 3D printer software by adjusting the build plate offset. Treating the coverslip with bind-silane eliminates the need for overexposed base layers, typically required for proper adhesion when printing on the build plate. This allows the channel to be printed open on the glass so that the coverslip forms one wall of the channel and the other three are defined by the cured resin. After printing was completed, the stack was removed from the build plate and sonicated in 99% isopropyl alcohol (IPA) for 1 min, followed by rinsing with IPA and deionized water (DIW). Compressed air was used to dry the print, and the microscope slide stiffener was left attached to the coverslip until the final assembly was complete.

The movable glass was made from a 700 μm thick borosilicate glass wafer (Borofloat 33, University Wafer, USA) diced into 3 mm × 4 mm rectangles with a Disco DAD 3221 dicing saw. After dicing, the individual glass pieces were attached to a single-sided water-soluble tape (5414, 3M, USA) to set the initial gap between the movable glass and the coverslip. The movable glass was placed into a designed cutout in the 3D-printed channel with water-soluble tape in contact with the coverslip. A custom-built dispenser was used to apply aquarium-grade silicone over the interface of the movable glass and the 3D-printed frame. Aquarium-grade silicone was chosen for this application owing to its high mechanical compliance and low chemical leaching. The alignment features on the 3D-printed channel frame enable the attachment of a separate 3D-printed structure, the control chamber, to the first 3D print using silicone and epoxy, centered over the movable glass. Adjusting the air pressure in the control chamber actuates the movable glass, changing the height of the fluidic channel. A 7 mm × 8 mm glass window was hand-scribed from a microscope slide and attached to the top of the control chamber using silicone, reinforced with epoxy, as shown in figure 2(a). 3D-printed barb fittings were threaded into the control chamber and sealed with silicone. The deformable channel’s inlet and outlet tubing (1/32 in. I.D, 3/32 in. O.D, Tygon E-3603) were press-fitted into openings in the 3D-printed channel frame, and silicone was used to secure them in place.

The water-soluble tape was removed by flushing the device with warm water. Fourier-transformed infrared spectroscopy was used to confirm the complete removal, as described in the Supplementary data and illustrated in figure SI.1. The last step before mounting on the microscope was to remove the microscope slide stiffener by using a heat gun to warm the microscope slide and activate the heat-release tape. Once the tape and microscope slide were removed, the assembly was clamped into a custom-built stiffener jig, as shown in figure 2(b), which kept the coverslip flat during imaging. The acrylic for the jig was laser-cut and assembled with double-sided tape and M3 hardware. A syringe pump, controlled by a custom LabVIEW VI, was used to regulate the pressure in the control chamber (figure 1 label A). This feedback-controlled pressure controller is described in the Supplementary data and shown in figure SI.2. The controller’s response time is shown in figure SI.3.

### 2.2. Cell culture

Bovine chondrocytes were harvested aseptically using a previously established protocol [43] from the stifle joints of 18–22 month old cattle obtained, from a local abattoir. The tissue was minced into ∼1 mm^3^ pieces and subjected to enzymatic digestion with Collagenase Type I (2 mg ml^−1^) (Gibco, Waltham, MA, USA) for 12–16 h at 37 °C in Dulbecco’s modified Eagle medium (DMEM) with 1% penicillin (10 000 I.U. ml^−1^), and streptomycin (10 000 μg ml^−1^) (Sigma, St. Louis, MO, USA) and gentle agitation on a continuous rotary shaker at 8–10 rpm. Isolated chondrocytes were cultured for 10 d in DMEM (Gibco, Waltham, MA, USA), buffered with 3.7 g l^−1^ sodium bicarbonate to maintain pH 7.2-7.4, supplemented with fetal bovine serum (FBS) (10% v/v) (Bio-Techne, Minneapolis, MN, USA), penicillin (10 000 I.U. ml^−1^), and streptomycin (10 000 μg ml^−1^) (Sigma, St. Louis, MO, USA) (hereby referred to as Complete media) in 5% CO2 at 37 °C and first passage population was used for experiments. For monolayer studies chondrocytes were cell cycle synchronized by 12 h starvation (Complete media lacking FBS) followed by culturing in Complete media for 48 h and incubated in 5% CO2 at 37 °C. Cells were trypsinized and cell viability was assessed using trypan blue exclusion (Gibco Trypan Blue solution, 0.4%). This method ensured a high yield of viable chondrocytes suitable for downstream applications. 3 × 10^6^ cells ml^−1^ of bovine chondrocytes were resuspended in phosphate buffer saline (PBS, 1x) and Calcein AM (eBioscienceTM Calcein Blue AM Viability Dye, Invitrogen) was added at a 5 μM final concentration. The cells were incubated in 5% CO2 at 37 °C in the dark for 30 min before cell compression studies.

### 2.3. Cell compression procedure

To reduce cell adhesion to the glass and 3D-printed channel, a 1% by volume solution of Pluronic F-127 (Sigma-Aldrich) in DIW was flown at 200 μl h^−1^ through the variable-height channel for 24 h before cell experiments, similar to [34]. The channels were flushed with 1x PBS before injecting cells suspended in 1x PBS with a syringe pump (GenieTouch, Kent Scientific, USA).

The cells were injected into the device until they were positioned underneath the movable glass and within the FOV, at which point the flow was stopped for the duration of the experiment. The movable glass plate was lowered by increasing the pressure in the control chamber until the cells were compressed. Several pressures, ranging from 2.5 psig to 3.5 psig, were chosen to vary the applied strain. Compression was maintained for 30 s for all experiments, and then the pressure in the control chamber was quickly lowered to raise the movable glass. The cells’ recovery was imaged for an additional 180 s after the compression was removed. Cells were imaged with a confocal microscope (Stellaris DMi8, Leica, USA) using a 20x objective (HC PL APO 20x/0.75 CS2, dry objective, Leica, USA). A time-series *Z*-stack was recorded at a 0.68 μm step height and 2.3 s stack frequency using the resonant scanning mode for improved frame rate. The *Z*-stack was used to guarantee that the maximum projected area of each cell was captured. The height of the *Z*-stack was less than the initial diameter of the cells, which improved the imaging frame rate.

To verify the uniformity of the channel in the FOV, the channel height was measured with the confocal microscope using reflectance imaging to find the glass–liquid interface. It was found that the variation in height did not exceed the *Z*-stack resolution of 0.63 μm, so further testing was not performed.

### 2.4. Image analysis

To calculate the total projected area (shadow) of the cells before, during, and after compression, each *Z*-stack of the time series was reduced to a single image using the maximum intensity projection (MIP) operation in Imaris (Oxford Instruments). The time series was then imported into MATLAB (Mathworks, USA), and each frame of the compressed time series was analyzed using a Sobel edge detection script to extract the total projected area of each cell during compression and recovery. The recovery curves of the individual cells were fitted using equation (3.4) using a non-linear least squares method in the Curve Fitting Toolbox of MATLAB to extract the relaxation time. Cells overlapping the edges of the FOV or in contact with other cells were excluded from the analysis.

## 3. Theory

**Figure 3.**
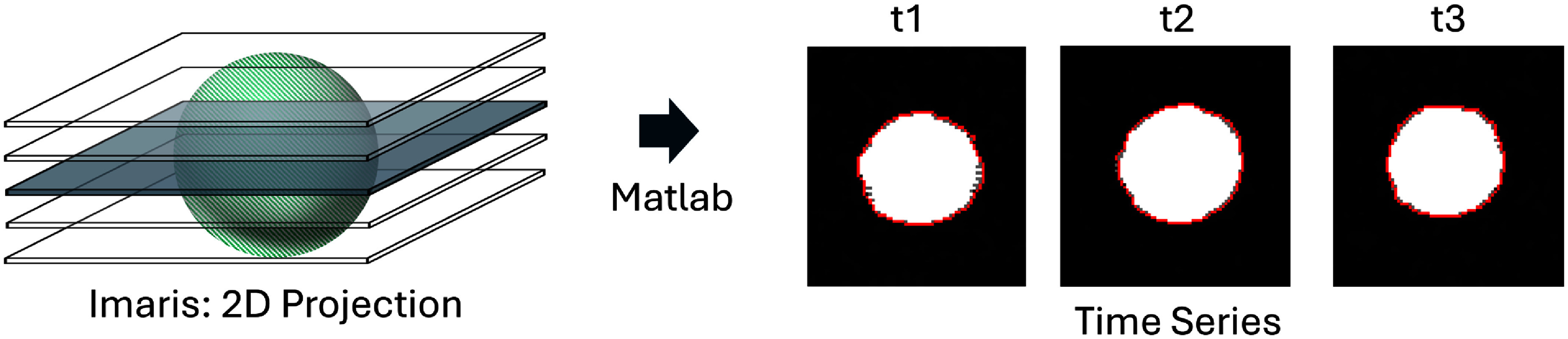
Each *Z*-stack in the time-series is converted to a single image by a MIP 2D projection in Imaris. Then the time-series is imported into Matlab and the projected area of each cell is found by an edge detection algorithm. The time series shows a single cell with the edge marked in red at different time points, *t*1, *t*2, and *t*3. Note that, for simplicity, only one cell is shown; however, there are more cells within the FOV.

The projected area shown in figure 3 is the measured parameter; however, it needs to be converted to fit an equivalent mechanical model. Therefore, it is converted to a linear strain by assuming that the cell is spherical prior to the applied strain and becomes an oblate spheroid during recovery, with *Rx* and *Ry* deforming at the same rate and magnitude as *Rz*, as described by [34]. In practice, the contact area between a cell and either of the glass plates is larger than an infinitesimal point, so the rigid spheroid assumption underestimates the linear strain of a cell compressed between two plates.

**Figure 4.**
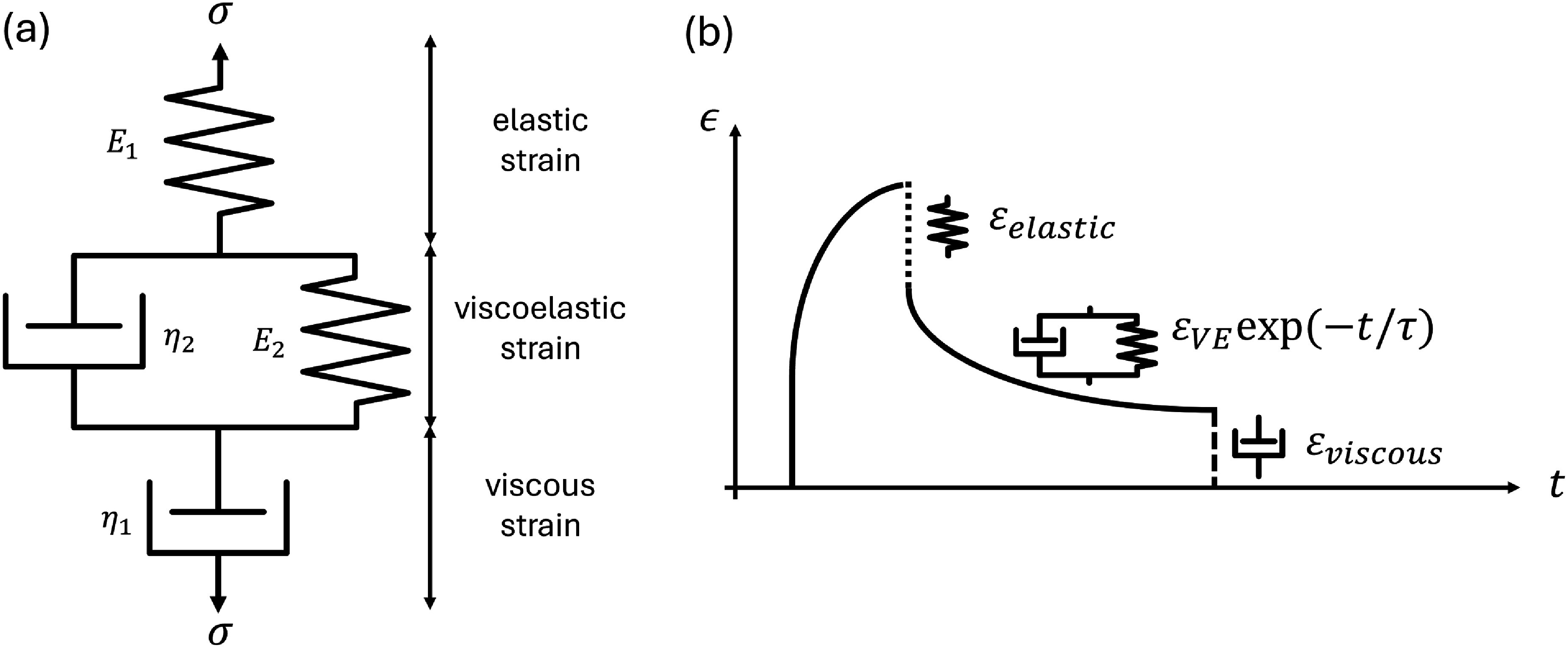
(a) Burgers model with the elastic, viscoelastic, and viscous strain labeled. (b) General recovery shape for an arbitrary strain. The viscoelastic strain takes the shape of an exponential function, and the total recovered strain is the sum of the elastic (${\varepsilon _{{\text{elastic}}}}$), viscoelastic (${\varepsilon _{{\text{VE}}}}$), and viscous (${\varepsilon _{{\text{viscous}}}}$) strains.

Viscoelastic models such as the SLS, also known as the Zener model (spring-dashpot series in parallel to another spring), has been applied to chondrocytes previously in MPA studies [11, 16, 44, 45]. However, this model fails to capture any permanent strain characteristics. Therefore, the four-element Burgers model (figure 4(a)) was used in this study. The viscoelastic recovery time is the key parameter measured in this work, and the SLS/Zener model could model the viscoelastic recovery and get an identical recovery time, so either the SLS or Burgers model would be adequate. As the strain magnitudes applied in this study were larger, up to 60%, the permanent strain was accounted for with the extra dashpot in the Burgers model and was the reason for choosing this model. This model is a series combination of the Maxwell (spring and dashpot in series) and Kelvin–Voigt (spring and dashpot in parallel) models. The constitutive equation for this model is,

\begin{align*} {\sigma + \left( {\frac{{{{{\eta }}_1}}}{{{E_1}}} + \frac{{{{{\eta }}_1}}}{{{E_2}}} + \frac{{{{{\eta }}_2}}}{{{E_2}}}} \right){{\dot \sigma }} + \frac{{{{{\eta }}_1}{{{\eta }}_2}}}{{{E_1}{E_2}}}\mathop {{\sigma }}\limits^{ \cdot \cdot } = {{{\eta }}_1}{{ \varepsilon }} + \frac{{{{{\eta }}_1}{{{\eta }}_2}}}{{{E_2}}}\mathop {{\varepsilon }}\limits^{ \cdot \cdot } }\end{align*}

where $\sigma $ is the stress, $\varepsilon $ is the strain, ${E_1}$ and ${E_2}$ are elasticity elements and ${\eta _1}$ and ${\eta _2}$ are viscosity elements. For a constant applied stress, the creep behavior is described as,

\begin{align*} {\varepsilon \left( t \right) = {\sigma _0}\left( {\frac{1}{{{E_1}}} + \frac{t}{{{\eta _1}}} + \frac{1}{{{E_2}\left( {1 - {{\text{e}}^{\left\{ { - \left( {{E_{2/{\eta _2}}}} \right)t} \right\}}}} \right)}}} \right)}\end{align*}

and for a constant applied strain, the stress relaxation is described as,

\begin{align*} {\sigma \left( t \right) = \frac{{{\varepsilon _0}}}{A}\left( {\left( {{q_1} - {q_2}{r_1}} \right){{\text{e}}^{ - {r_1}t}} - \left( {{q_1} - {q_2}{r_2}} \right){{\text{e}}^{ - {r_2}t}}} \right)}\end{align*}

where $A = \sqrt {p_1^2-4{p_2}} $, ${q_1} = {\eta _1}$, ${q_2} = \frac{{{\eta _1}et{a_2}}}{{{E_2}}}$, ${p_1} = \frac{{{\eta _1}}}{{{E_1}}} + \frac{{{\eta _1}}}{{{E_2}}} + \frac{{{\eta _2}}}{{{E_2}}}$, and ${p_2} = \frac{{{\eta _e}t{a_2}}}{{{E_1}{E_2}}}$. Both forms of unconstrained recovery, where the applied stress and strain are zero, take the same general form shown in figure 4(b),

\begin{align*} {\varepsilon \left( t \right) = {\varepsilon _{{\text{elastic}}}} + {\varepsilon _{{\text{VE}}}}{\text{exp}}\left( {\frac{{ - t}}{{{\tau }}}} \right) + {\varepsilon _{{\text{viscous}}}}}\end{align*}

where $\tau $ is the viscoelastic recovery time, and the ${\varepsilon _{{\text{elastic}}}},{ }{\varepsilon _{{\text{VE}}}},{\text{and }}{\varepsilon _{{\text{viscous}}}}$ terms describe the instantaneous elastic recovery, the constant of exponentially decaying recovery, and the permanent strain due to the viscous flow of the dashpot ${\eta _1}$ [46].

## 4. Results and discussion

### 4.1. Image analysis and curve fitting

Although a threshold edge detection method was initially attempted, the position of the cell edge proved inconsistent cell-to-cell due to the varying fluorescent intensities of the cells. The Sobel edge detection method is based on the intensity gradient, which results in a more consistent cell edge when analyzing cells with varying intensities.

**Figure 5.**
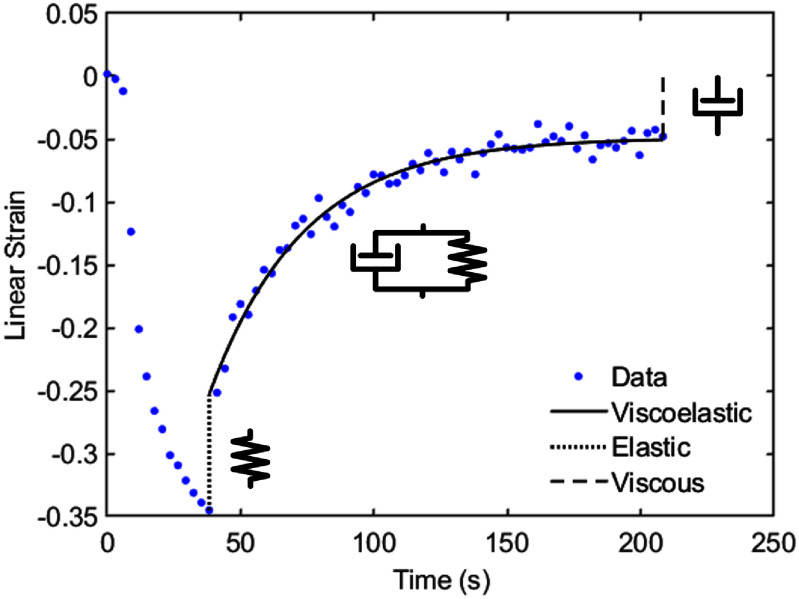
The converted linear strain of a typical cell during an experiment with the fitted elastic, viscoelastic, and viscous recovery strains labeled. The cell has no strain prior to compression, then experiences an increasing compressive strain from a constant applied pressure from the top glass plate, and finally relaxes towards its undeformed shape. The viscous element in the Burgers model account for any permanent strain.

An example of the measured projected area of a cell and the calculated linear strain over time is shown in figure 5. The cell is initially unstrained before a load is applied, where the cell continuously deforms as is typical in a constant stress experiment. After 30 s, the applied stress is reformed from the cell and the cell shows rapid elastic recovery, followed by a viscoelastic response. Any unrecovered strain is accounted for as permanent strain.

### 4.2. Cell deformation and recovery

**Figure 6.**
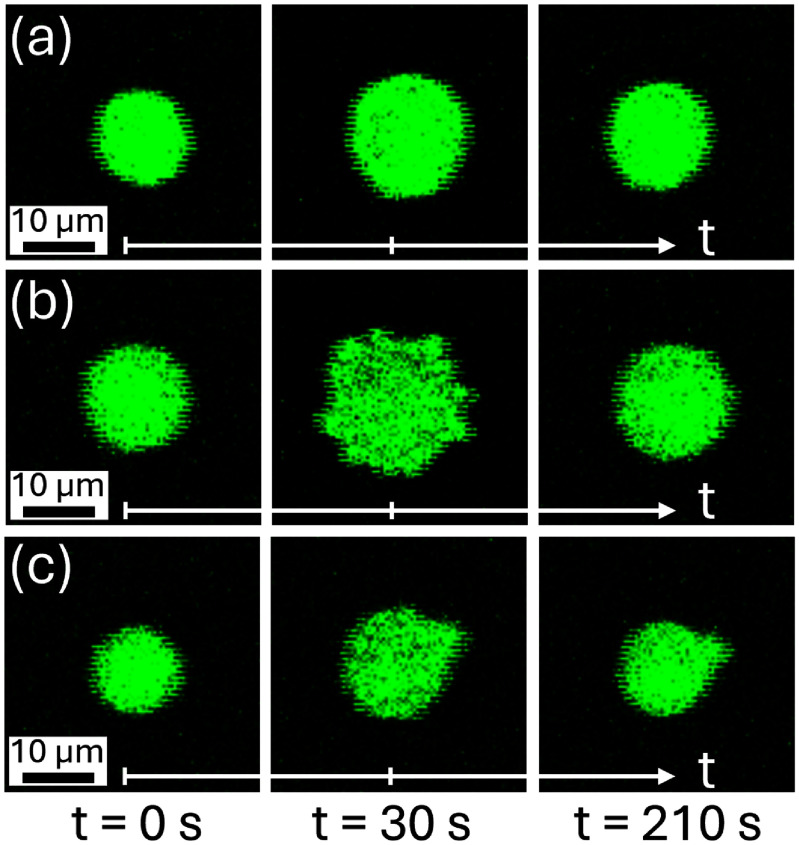
Images of three cells at three time points; prior to applied strain, at maximum strain, and after 3 min of recovery, respectively. Examples of (a) radially-symmetric deformation, (b) temporary radially-asymmetric cell membrane deformation during applied strain, and (c) permanent radially-asymmetric deformation. Note that these images were captured using resonant scanning mode which decreases quality but drastically improves frame rate.

The typical response of the bovine cells strained in these experiments is radially-symmetric deformation in the transverse plane, where the projected area, shown in figure 6(a), increases and then decreases with the application and removal of the strain, respectively. However, some cells studied in this experiment exhibited both short-term and long-term radially-asymmetric deformation. The short-term radially-asymmetric deformation recovered within 3 min, whereas the long-term effects persisted longer than the cells were imaged. This radially-asymmetric deformation is shown in figures 6(b) and (c).

**Figure 7.**
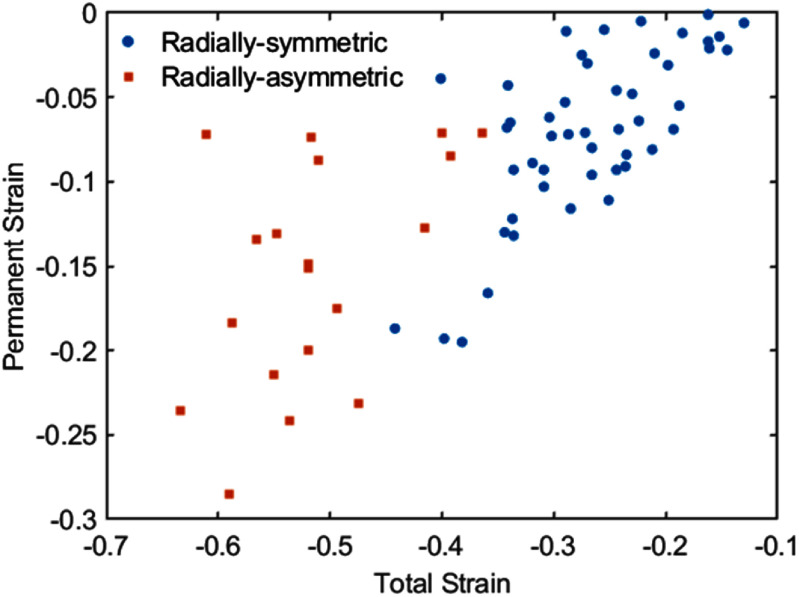
Total vs permanent strain of cells. Cells with radially-symmetric deformation are represented by blue circles (*n* = 48), while cells exhibiting short-term radially asymmetric deformation are depicted as red squares (*n* = 19).

Compressive strains in the range of 10%–60% were applied, and it was found that more than 40% of the cells exhibited short-term radially-asymmetric membrane deformation, as shown in figure 7. The cells that experienced temporary radially-asymmetric membrane deformation recovered their original spherical shape, although not necessarily their original diameter. Generally, the resulting permanent strain increased with the magnitude of the applied strain.

**Figure 8.**
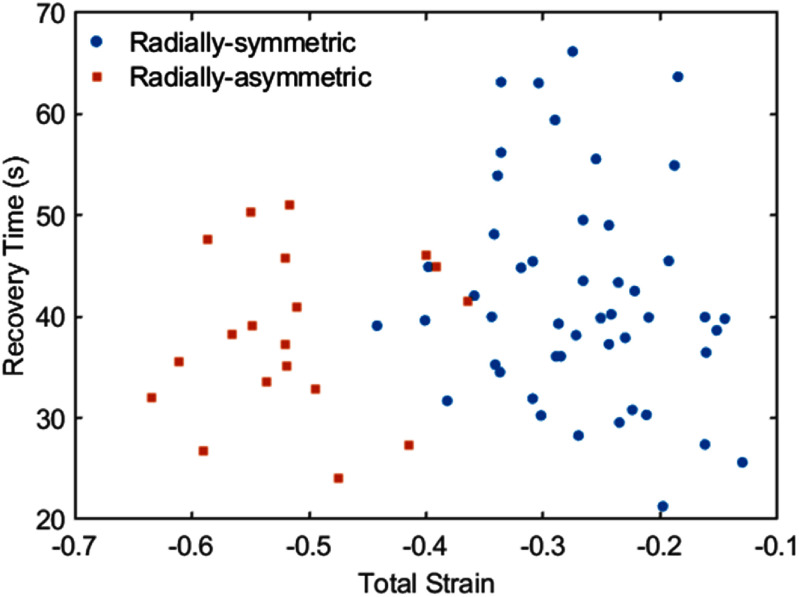
Recovery time of bovine chondrocytes as a function of total applied compressive strain. The blue circles represent the radially-symmetric deformed cells (*n* = 48) while the red squares are cells (*n* = 19) which experienced short-term radially-asymmetric deformation.

The viscoelastic recovery time of radially-symmetric bovine chondrocytes was an average of 42 s with a standard deviation of 11 s (*n* = 48). The recovery time did not depend on the magnitude of the applied strain, as shown in figure 8. Additionally, cells with radially-asymmetric deformation recovered similarly to cells with radially-symmetric deformation, with a mean of 38 s and a standard deviation of 8 s (*n* = 19). The recovery times did not strongly depend on the applied strain.

### 4.3. Discussion

The recovery time of chondrocytes that displayed radially-symmetric and radially-asymmetric deformation were not statistically significantly different, with mean recovery times of 42 s and 38 s, respectively, which is in agreement with previous MPA and AFM characterization in [38, 40, 41, 47–49] and listed in table 1. The MPA studies used a three-element Zener mechanical model, which is different from the model used in this study. The Burgers model was chosen for this work as it accounted for the permanent deformation of the cells. Chondrocytes from humans, pigs, and rabbits are reported, while bovine chondrocytes are used in this study. The recovery times found in this work are reasonable when compared to those reported, indicating the validity of the findings.

An applied strain magnitude greater than 40% appeared to demarcate radially-symmetric and radially-asymmetric deformed cells. This radially-asymmetric deformation was only a temporary phenomenon, and the cells from this dataset all recovered to their original circular shapes. In cases where the strain magnitude exceeded 40%, approximately 20% of cells exhibited radially asymmetric deformation, which was not recovered within the experiment time, and the recovery time was not measured. It was determined that the permanent strain was independent of cell size, as shown in figure SI.4.

One explanation for the radially-asymmetric cell deformation shown in figure 6(b) is blebbing. Blebbing is a process in which the plasma membrane of a cell forms spherical protrusions or bulges. Blebbing can be a normal physiological response, such as during cell movement [39, 50] and division [51], or it can be associated with pathological conditions like apoptosis [52, 53]. Blebs typically form in 30 s before shrinking over 120 s and reaching a size of approximately 2 μm [54].

Blebbing has been observed during MPA in various cells, including chondrocytes [55–58]. Sliogeryte *et al* found a critical pressure threshold where bovine chondrocytes began blebbing in MPA experiments [57]. The results of this study reveal a similar phenomenon: chondrocytes subjected to a critical linear strain of over 40% exhibit short-term radially-asymmetric deformation. This suggests that the radially-asymmetric deformation observed in this study is cell blebbing. Although briefly discussed here, cell blebbing quantification is outside the scope of the present work. Sliogeryte *et al* attribute cell blebbing to membrane-actin cortex detachment and concluded that chondrocyte dedifferentiation increases cortical F-actin organization and affected the critical pressure threshold [58]. Additional work would be required to investigate this phenomenon, such as live-cell imaging with cytoskeletal markers.

Treating the cell as an isotropic viscoelastic solid and assuming to cell deformation as a rigid spheroid is a large oversimplification. It was done to simplify the use of the device and analysis of the data. These assumptions have been previously made in a similar PDMS-based device [34]. A potential improvement is to estimate the contact area using the Hertzian theory for non-adhesive elastic contact between a sphere and a flat plate. Using a larger and finer *Z*-stack CLSM scan could provide data on the contact area and total cell shape. Assuming the cell as an isotropic material limits the results to bulk cell characteristics.

There are several ways in which this method could be significantly improved. The cell suspension concentration and imaging FOV are the two factors that limit imaging throughput. The cell concentration in this study could be optimized to increase the number of cells visualized in parallel. One challenge with a higher cell concentration is that it increases the likelihood of cells coming into contact with each other, making image analysis more difficult. For simplicity, this study did not investigate any cells in contact with other cells. While 3 × 10^6^ cells ml^−1^ was found to be a good starting point, resulting in roughly 20 cells in the FOV, the cells tend to fall out of suspension over time. Adding a stir bar mixer in the syringe with cells should be considered in the future. The FOV in this study was 0.20 mm^2^, defined by the microscopy system. As currently designed, the maximum FOV is 12 mm^2^, defined by the size of the movable glass, which is 60x larger than the FOV of this study. As a rough estimate, assuming a cell occupies a 100 μm × 100 μm region and the maximum FOV is employed, then 1200 cells could be tested in parallel. If a larger FOV microscopy system could be utilized, adding rigid glass or polystyrene microspheres to the cell suspension would enable a fluidic chamber of constant height capable of compressing all cells within the FOV. The strain magnitude applied to each cell would depend on the individual cell diameter compared to the microspheres. Currently, the applied strain is dependent on the pressure in the control chamber and the number of cells under the movable glass plate. Due to fabrication variation, the pressure required to apply a desired strain to the cells varies for each device. Using microspheres to set the height would eliminate this variation, but it would also introduce the complexity of flushing different-sized microspheres in and out to achieve the desired applied strain magnitude. Along with these potential improvements, precise control of the fluid in which the cells are suspended is desirable to efficiently and effectively replace all the cells under the movable glass plate and control the horizontal compression.

The glass surfaces used to compress the cells in the device were treated with Pluronic F-127 to reduce cell adhesion. Without this modification, many cells stuck to one or both glass surfaces, so they did not recover naturally. Additionally, after recovery, some cells adhered to the glass, even when flow was applied to position new cells between the glass plates within the FOV. Du *et al* also treated their microfluidic device with Pluronic solution and found that the recovery times of human promyelocytic leukemia HL60 cells and 3T3 fibroblast cells were statistically and not-statistically significantly altered, respectively [34]. Further work is required to investigate if the surface treatment modifies cellular mechanical properties in any way beyond reducing cell-glass adhesion.

The experiments were done in a non-climate-controlled environment for simplicity. Ideally, cells would be kept at 37 °C during experiments as viability likely affects cell mechanics. However, cells were stained and imaged with a fluorescent viability dye to confirm that cells were living during imaging. Dim cells were not investigated. Additionally, the use of a syringe pump to control the pressure in the control chamber could be improved by using a dedicated air pump. As it stands, the measured response time is significantly faster than the imaging frame rate, so the introduced error is negligible. If the pressure value were used to extract cell stiffness, then more precise control would be required.

The previously described assumptions and challenges collectively contribute to measurement variability. Additionally, the cell orientation during compression may add variability, as the cell properties are not guaranteed to be symmetric. The edge detection is sensitive to cell brightness and thresholding inputs, so it is possible that it over- and under-estimates cells’ projected areas to some extent. Taking all these sources of variation into account, it is still reasonable that there exists cell-to-cell variation in mechanical properties, as evidenced by the range and uncertainty of previously reported cell characterization.

As an extension of this study, multiple compression times should be investigated, as the recovery time is dependent on it. Additionally, the hysteresis of cell recovery time could be investigated by applying multiple compression-recovery experiments to the same group of cells. This is currently feasible with the current device and experimental methods. Another aspect to explore is applying time-dependent strain to simulate the typical cyclical compression of articular cartilage.

There are numerous potential applications for using this device to characterize the viscoelastic properties of cells. It has been demonstrated that breast cancer cells exhibit distinct mechanical properties, e.g. lower stiffness, compared to healthy epithelial cells, and that cancerous cells are softer than cells from non-malignant or less-differentiated cancers [17, 59]. Beyond the study of biomechanics, the viscoelastic characterization of oral cancer cells is an example of a possible application of this device as a diagnostic tool for detecting malignant change [60]. Similarly, increased red blood cell deformability has been associated with diabetic foot disease and sickle cell disease [61, 62]. Finally, the elastic modulus of chondrocytes and chondrocyte PCM from healthy and OA cartilage may be altered with the onset and development of OA [41, 63, 64]. The methodology can be directly applied to study chondrocytes from OA cartilage to explore how viscoelastic properties are altered compared to those of their normal counterparts.

## 5. Conclusion

The device developed in this work demonstrates a reproducible and accurate ability to deform spherical cells in a manner compatible with microscopy. The projected area of the deformed cells was imaged using confocal microscopy and measured using an edge detection algorithm. This projected area was fit to a Burgers viscoelastic model, and the elastic, viscoelastic, and permanent strains were measured. The measured viscoelastic recovery times of bovine chondrocytes were consistent with those obtained from previous MPA characterization. While the recovery times did not depend on the applied strain, the resulting permanent strain increased in magnitude with the larger applied strain.

Radially-asymmetric deformation of cells was observed for strains above approximately 40%. This phenomenon was argued to be cell blebbing, as similar results have been observed in chondrocytes during MPA. Further work using this device could determine recovery time dependence on compression time, as the current experiments fixed this time at 30 s. Additionally, a more robust conversion of the projected area to linear strain could be attempted to determine the strain applied to the cells more accurately. While chondrocytes were used in this work, these methods could be adapted for other types of spherical cells.

The primary advantage of this device over MPA or other techniques is its ability to simultaneously strain multiple cells. The number of cells characterized in parallel is determined by the FOV of the imaging system, assuming an appropriate cell concentration is used. In this work, the FOV was 450 × 450 μm. However, this system can be adapted to other microscopy systems to increase the FOV and, consequently, the overall characterization throughput. The device is cost-effective and accessible to many researchers because it is primarily fabricated through 3D printing rather than requiring PDMS mold fabrication facilities.

## Data Availability

The data cannot be made publicly available upon publication because no suitable repository exists for hosting data in this field of study. The data that support the findings of this study are available upon reasonable request from the authors.
